# RGCA: A Reliable GPU Cluster Architecture for Large-Scale Internet of Things Computing Based on Effective Performance-Energy Optimization

**DOI:** 10.3390/s17081799

**Published:** 2017-08-04

**Authors:** Yuling Fang, Qingkui Chen, Neal N. Xiong, Deyu Zhao, Jingjuan Wang

**Affiliations:** 1 University of Shanghai for Science and Technology, Shanghai 200093, China; forwardfyl@163.com (Y.F.); xiongnaixue@gmail.com (N.N.X); zhao781201@163.com (D.Z.); wjj9209@163.com (J.W.); 2 Department of Mathematics and Computer Science, Northeastern State University, Tahlequah, OK 74464, USA

**Keywords:** Internet of Things, data mining algorithms, GPU cluster, performance, energy consumption, reliability

## Abstract

This paper aims to develop a low-cost, high-performance and high-reliability computing system to process large-scale data using common data mining algorithms in the Internet of Things (IoT) computing environment. Considering the characteristics of IoT data processing, similar to mainstream high performance computing, we use a GPU (Graphics Processing Unit) cluster to achieve better IoT services. Firstly, we present an energy consumption calculation method (ECCM) based on WSNs. Then, using the CUDA (Compute Unified Device Architecture) Programming model, we propose a Two-level Parallel Optimization Model (TLPOM) which exploits reasonable resource planning and common compiler optimization techniques to obtain the best blocks and threads configuration considering the resource constraints of each node. The key to this part is dynamic coupling Thread-Level Parallelism (TLP) and Instruction-Level Parallelism (ILP) to improve the performance of the algorithms without additional energy consumption. Finally, combining the ECCM and the TLPOM, we use the Reliable GPU Cluster Architecture (RGCA) to obtain a high-reliability computing system considering the nodes’ diversity, algorithm characteristics, etc. The results show that the performance of the algorithms significantly increased by 34.1%, 33.96% and 24.07% for Fermi, Kepler and Maxwell on average with TLPOM and the RGCA ensures that our IoT computing system provides low-cost and high-reliability services.

## 1. Introduction

Since the first day it appeared, the Internet of Things (IoT) [1,2] has been regarded as a technology for seamlessly connecting traditional networks and networked things [3]. The major idea of IoT is to connect the Internet to all objects used by us in the world [4]. Nowadays, researchers from all walks of life are focused on revolutionizing the Internet [5] by structuring a more convenient environment that is made up of various intelligent systems, such as smart homes, intelligent healthcare, global supply chain logistics, etc. [6]. The domestic and international applications of the IoT mainly include smart homes, intelligent environmental protection, intelligent transportation, intelligent security and industrial monitoring, etc. In this paper, we pay attention to the application of IoT for smart homes.

With the development of modern technology, people are no longer satisfied with the fundamental functions of their houses, and seek more social functions, such as home offices, online education and information about all the places [7]. Smart homes are built through the use of wireless sensors, image recognition, RFID, positioning and other technical means to comprehensively perceive the family environment and changes to people and things [8]. The establishment of monitoring and early warning systems in the family home through Wireless Sensor Networks (WSNs) based on the IoTs build up informatization and intellectualization remote control networks. so that users of the system can clearly and in a timely fashion understand the current state of the environment [9]. However, it is necessary to establish an efficient and high-reliability model to guarantee that the system can perform continuously without malfunction. In the work in [10,11,12], an energy conserving mechanism and a high-energy efficient scheme for the IoT were studied. The energy consumption was also discussed in [13,14], and the researchers used genetic improvement to reduce energy consumption to a large extent.

**Figure 1:**
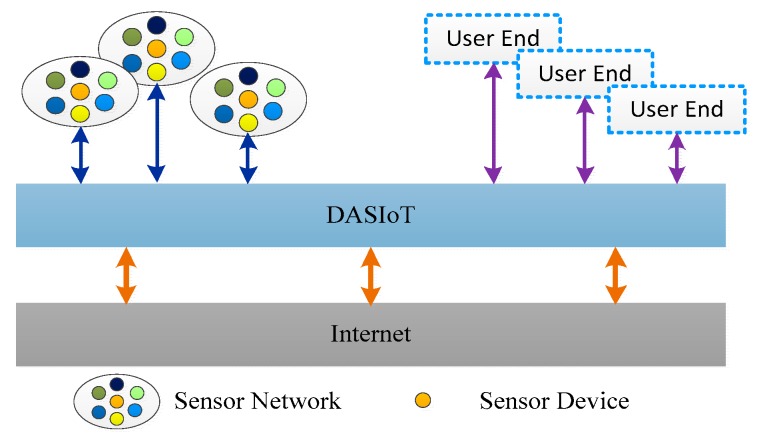
Structure of the integrated IoT system.

Our large-scale smart home cloud platform lab can access and store 48 M data per second in real-time from 420,000 households, enabling millions of bytes of data throughout per second. In IoT, the massive amount of data generated by a user’s family is collected by the sensor network deployed in each home, and the collected data packets are decoded and then transformed in the Data Access System for the Internet of Things (DASIoT) [15]. After DASIoT’s decoding and transformation, these data are transformed and communicated for the automatic detection, identification, tracking and control of the objects [16] and execution of the application system so that an intelligent network is established between humans, the Internet and things. In terms of the framework of the IoT application system, DASIoT mainly works at the application level of the IoT, as shown in Figure 1.

In Figure 1, the main function of the DASIoT is to provide data access services for end users and issue the large amount of requests from users by transmitting them on to the sensor devices. For data processing, there is a problem that cannot be ignored. How do we transform the data collected by DASIoT into useful information to provide a better service for people? This is where useful information discovery in database and data mining technologies comes into play, for these technologies provide solutions to mining information hidden in the data of IoT which can be used to improve the performance or energy consumption of the computing system [17]. Previous researchers focused on using or developing effective data mining technologies for the DASIoT, and their results in [18,19] prove that some data mining algorithms are able to make IoT more useful and intelligent, thus providing faster and smarter services.

In this paper, we not only give a related description of common data mining algorithms in IoT computing but also propose a detailed performance-energy optimization scheme based on a GPU (Graphics Processing Unit) cluster in order to acquire better services. In this study, we answer the following important questions: Is the CUDA (Compute Unified Device Architecture) programming model suitable for common data mining algorithms in IoT computing? Can the GPU cluster help IoT achieve a low-overhead, high real-time and high-reliability system? In addition to adjusting Thread-Level Parallelism (TLP), what can be done to improve the performance of parallel programs? To achieve these goals, we propose a new optimization architecture for IoT computing which is applicable, accurate and straightforward for different data mining algorithms. It is worth mentioning that, unlike previous performance-energy models, our model applies to different types of applications, including compute-bound, memory-bound and balanced type kernels. By exploiting the appropriate TLP and instruction-level parallelism (ILP) configuration, the Two-level Parallel Optimization Model (TLPOM) improves its average performance by 34.1%, 33.96% and 24.07% for Fermi, Kepler and Maxwell architectures, respectively. Then, on the basis of considering the type of kernels, we use different computing nodes in our cluster. Finally, we obtain a low-cost and high-reliability computing system.

The rest of this paper is organized as follows: in Section 2, we introduce the related work. Section 3 presents the energy consumption calculation model and our current sampling method. The TLPOM is presented in Section 4 and Section 5 gives our RGCA (Reliable GPU Cluster Architecture) for the high-reliability computing system. Our experiment results and analysis are shown in Section 6. Finally, we conclude this paper in Section 7.

## 2. Related Work

**Figure 2:**
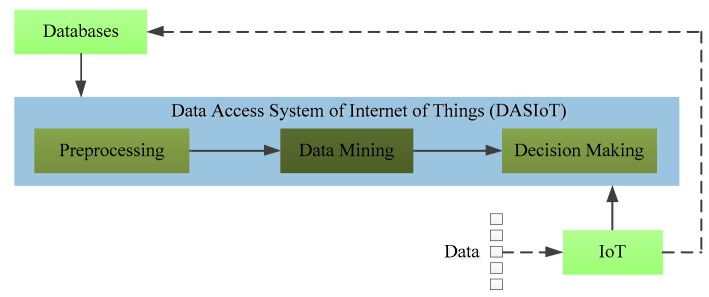
Basic structure of the DASIoT.

With the development of the IoT, an increasing amount of large-scale data needs to be processed in the DASIoT. However, challenges concerning how to process the data and how to extract useful information have emerged in recent years. The problem we have to consider is that the data from IoT is usually too large and it is too difficult to process using the ways available today [20,21]. As paper [22] proposed, the bottleneck of IoT services will shift from Internet to data mining, transformation, etc. Since the issue of processing massive data has been studied for years, it is not surprising that some classical but practical ways have been applied in IoT, such as random sampling [23], data condensation [24], and incremental learning [25]. However, these methods only handle partial data instead of all the data, so all of these studies need a data preprocess in DASIoT, as shown in Figure 2. In addition to preprocessing, how to obtain useful information from these data has become a crucial issue for better IoT services. According to [17], the data mining technique is responsible for extracting information from the output of the last step and then delivering this into the next step, which converts its input into useful information. Therefore, in relation to big data, the application of data mining techniques has become increasingly extensive [18,26].

### 2.1. Introduction to Common Data Mining Algorithms

It is much harder to analyze data than to collect data. In order to achieve a high-performance and high-reliability data mining component for IoT computing, there are several critical factors when choosing applicable mining technologies: the objective, input data, output data, the characteristics of data, application scenario and mining algorithm. According to [17,18], data mining algorithms can be divided into many categories based on their application, including classification, clustering, association analysis, time series, outlier analysis, etc. Here we briefly introduce several common data mining algorithms and their features:

- k-means: As one of the most well-known clustering algorithms, k-means [27] is widely used in multiple domains, such as data mining and pattern recognition. It generally analyzes data objects without consulting a known class model. Firstly, a user-specified number of clusters, k, is assigned randomly to represent the number of the input patterns in different groups. Then, the assignment operator computes the minimization of intra-cluster distance and the maximization of the inter-cluster distance, and this idea complies with parallel reduction, so a high-efficiency parallel k-means algorithm should be considered for better performance.
- KNN: This is one of the most traditional and simplest algorithms in data mining, k-nearest neighbor (KNN) [28], which was proposed by Steinbach. It memorizes the entire training data and performs classification only if the attributes of the test object match one of the training examples exactly. KNN provides a summary of the nearest-neighbor classification method. It calculates the distance between test data and all the training data to determine its nearest-neighbor list. Once the list is finished, the test object can be classified.
- BPNN: The back-propagation neural network (BPNN) is a multilayer negative feedforward neutral network trained by the error back propagation algorithm, which is the most widely used neutral network. It has the ability of non-linear mapping and parallel distributed processing, as well as being self-learning and adaptive. It also allows an arbitrary number of inputs and outputs. BPNN is also one of the common classification algorithms in IoT computing [29]. In addition to classification, it can also be used to predict conditions in the home environment, such as temperature and humidity. According to its algorithm structure, we know that BPNN offers plenty of operations which are suitable for matrix multiplication, dot product, etc., and this provides the basis for the parallel optimization of BPNN.
- SVM: Support Vector Machine (SVM) is a kind of supervised learning method. It is widely used in data applications, for example data mining [17,28] in the IoT. The basic idea of SVM is to map the vector into high-dimensional space, and then to establish the vector spacing of the largest hyperplane to separate these data. SVM involves three phases: learning phase, cross-validation phase and classification prediction phase. While in the learning phase, it can consume a large amount of computational resources, therefore, how to enhance the efficiency of processing large-scale data is the major issue.
- GMM: Gaussian mixture model (GMM) is also a clustering algorithm used in IoT. Unlike k-means, GMM gives the probability that data points are assigned to each cluster [30], also known as soft assignment. In some applications, the probability has a great effect, for example disease diagnosis. Just as its name implies, it assumes that the data follows the mixture Gaussian distribution. In other words, the data can be thought of being generated from several Gaussian distributions. In addition, by increasing the number of models, we can arbitrarily approximate any continuous probability dense distribution, and each GMM consists of k Gaussian distributions.

By combining the above algorithms and their basic functions, we extract the benchmarks to optimize our parallel optimization method, as shown in Table 1. All these algorithms have been paralleled in previous research [31], and we have also modified the parallelism. Reduction is a class of parallel algorithms which produces one result using a binary operator ⊕ that conforms to the associative law. Such operations include minimization, maximization, sum, squared sum, logic and/or vector dot product, etc. This is an important basic step in other advanced algorithms, such as k-means, KNN and GMM. Matrixmul is used in many data mining algorithms, such as SVM, spectral clustering and PCA (Principal Component Analysis). The Gaussian kernel function is the most commonly used radial basic function (RBF) which has several important properties, such as rotational symmetry, separability, the single spectral Fourier transform, etc. These features indicate that the Gaussian smooth filter is a very effective low-pass filter in both the frequency domain and time domain. It is widely used for RBF, BPNN and GMM. The FFT (Fast Fourier transform) is also an important basic algorithm in many fields. For example, it is used for signal processing [32] in data mining.

### 2.2. CUDA Programming Model and GPU Architecture

#### 2.2.1. CUDA Programming Model

The Compute Unified Device Architecture (CUDA) is a general parallel computing architecture released by NVIDIA (Santa Clara, USA). It enables applications to take full advantage of the respective merits of the CPU and GPU, and solve complex computing problems. In CUDA, the CPU is regarded as the host and GPU as the coprocessor or device, and they work cooperatively to perform their functions. The CPU is responsible for logical transaction processing and serial computing, and the GPU focuses on highly linearized parallel task processing [33]. They each have their own independent memory address space: host memory and device memory.

**Figure 3:**
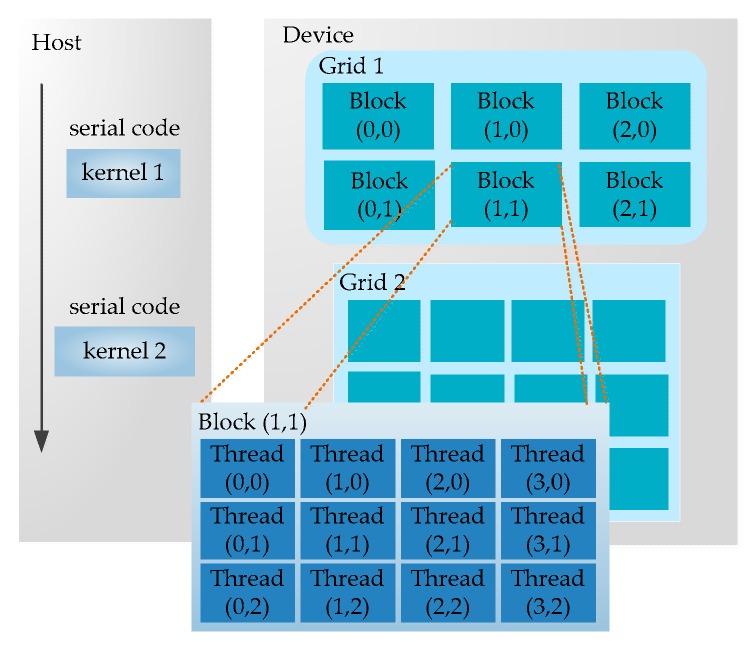
CUDA programming model.

Once the kernel is determined, this part is handed over to the GPU. The parallel computing function running on the GPU is called the kernel. A kernel is just a part of a program, and it can be executed in parallel. As shown in Figure 3, a complete CUDA program consists of multiple device-end parallel kernels and the host-end serial processing steps. There are two-level parallel hierarchy in a kernel, and one is the blocks in the grid and another is the threads in the block [33]. The two-tier parallel model is one of the most important innovations of CUDA, which is also the original source of inspiration for our performance optimization model (in Section 4).

#### 2.2.2. GPU Architecture

**Figure 4:**
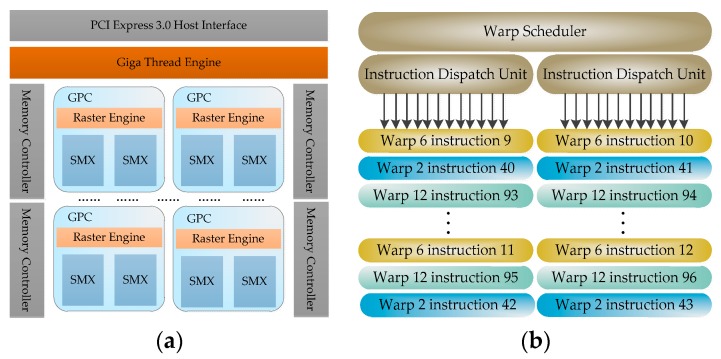
GeForce GTX 680 main architecture: (**a**) Block diagram; (**b**) A single warp scheduler.

NVIDIA and AMD (Silicon Valley, CA, USA) are the principal suppliers of civilian and military graphics cards. In this paper, we use three kinds of GPUs: Fermi, Kepler and Maxwell. For simplicity, we mainly focus on the Kepler GTX 680 (NVIDIA, Santa Clara, CA, USA) (called simply 680 from hereon in) GPU in this section. Large-scale parallelism in GPU hardware is achieved by repeatedly setting up multiple identical general building blocks (SMs). Figure 4a shows an overview of the 680 block diagram. GPU receives CPU commands via the Host Interface. The GigaThread Engine [34] creates and dispatches blocks to SM, and manages the context switches between threads during execution. An individual SM in turn schedules, warps and maps the threads to the CUDA cores and other execution units (LD/ST, SFU). The basic unit of execution flow in the SM is the warp, which contains 32 parallel threads and executes in a single instruction multiple thread (SIMT) [33] model. Using GPU, one of the key aspects for applications is breaking down the kernels into appropriate-sized grids and blocksize, where the grid indicates the dimension of the thread block and the blocksize indicates the number of threads per thread block. A bad choice of thread layout typically also results in a bad memory pattern, which will strikingly hurt performance [35]. GPUs always utilize high TLP to cover the processing latency of different warps [36].

From Kepler, each SM contains four warp schedulers and eight instruction dispatch units, as shown in Figure 4b, allowing four warps to be issued and executed concurrently. Having less than four warps means that some instruction dispatch units will be idle, effectively hurting the instruction dispatch speed. Another important cause of performance constraint is shared resource usage. Resource contention limits the ability of shared resources (including CUDA cores, load/store unit, registers, shared memory, etc.) to continuously perform the same operations. According to the launch configuration [35], we try to optimize it in the following aspects: the number of threads per block, the number of blocks and the tasks performed per thread (ILP). Therefore, in this paper, we combine TLP and ILP to improve performance and reduce energy consumption.

## 3. Power Consumption Calculation Model

### 3.1. Traditional Power Consumption Model

According to the CPU power consumption calculation definition [37], GPU power consumption consists of three parts: dynamic power consumption, short-circuit power consumption and power loss due to transistor leakage currents:

(1) $$P_{GPU}=P_{dynamic}+P_{static}+P_{leak}$$

In Equation (1), $P_{dynamic}$ is from the activity of logic gates inside a GPU, and $P_{static}$ is mainly determined by the device itself. Both $P_{dynamic}$ and $P_{static}$ are dependent on the clock frequency, while $P_{leak}$ is dependent on the GPU supply voltage.

### 3.2. Our Power Consumption Model

The power consumption model proposed in this paper is different from using a power analyzer to directly measure the power consumption of GPU, as we calculate the energy consumption of a computing node according to the work in [38]. The energy consumption $E_{cn_{i}}$ of the *i*th computing node *cn_i_* at the GPU cluster can be calculated by power consumption $P_{cn_{i}}(I_{i}(t))$ during time interval $\left[t_{0},t_{n-1}\right]$, as shown in Equation (2):

(2) $$E_{cn_{i}}=\int_{t_{0}}^{t_{n-1}}P_{cn_{i}}(I_{i}(t))d_{t}$$

where power consumption $P_{cn_{i}}(I_{i}(t))$ can be represented by Equation (3):

(3) $$P_{cn_{i}}(I_{i}(t))=P_{cn_{i}}^{idl}(I_{i}(t))+P_{cn_{i}}^{dny}(I_{i}(t))$$

where $P_{cn_{i}}^{idl}(I_{i}(t))$ is the power consumption when the *i*th computing node is in the idle state, $P_{cn_{i}}^{dny}(I_{i}(t))$ is the power consumption spreading from idle to full utilized, and $I_{i}(t)$ is the current on the *i*th computing node, which changes over time. We present the measurement method in Section 3.3.

According to [39] and Section 3.1, power consumption $P_{cn_{i}}^{idl}(I_{i}(t))$ is mostly determined by the GPU state, the number of active CUDA cores and memory type etc. The power consumption of the application of DVFS [40] on the GPU is almost a linear relationship for a computing node., so:

(4) $$\left\{ \begin{array}{l} P_{cn_{i}}^{idl}(I_{i}(t))=U_{cn_{i}}\cdot I_{idl}\\ P_{cn_{i}}^{dny}(I_{i}(t))=U_{cn_{i}}\cdot i_{cn_{i}}^{dny}(t) \end{array}\right.$$

where $I_{idl}$ is the static current of per node, it is a constant, while $i_{cn_{i}}^{dny}(t)$ is the working current per node and is determined by the applications and the specific node.

The energy consumption $E_{cn_{i}}$ of the node can be further expressed by Theorem 1.

**Theorem** **1.** *If the time interval* $\left[t_{0},t_{n-1}\right]$ *can be further divided into* $\left[t_{0},\;t_{1},\;\ldots ,\;t_{n-1}\right],\;\forall \left[t_{k-1},t_{k}\right],\;k\;\in \left[1,\;2,\;\ldots ,\;n\right]$*, and* $i_{cn_{i}}^{dny}(t\in \left[t_{k-1},\;t_{k}\right])\equiv i_{ik}$*, where* $i_{ik}$ *is a constant, then energy consumption* $E_{cn_{i}}$ *can be expressed by Equation (5),*

(5) $$E_{cn_{i}}=U_{cn_{i}}\cdot I_{idl}\cdot (t_{n-1}-t_{0})+U_{cn_{i}}\cdot \sum_{k=1}^{n-1}\left(i_{i_{k-1}}\cdot \left(t_{k}-t_{k-1}\right)\right)$$

**Proof.**  The power consumption $P_{cn_{i}}(I_{i}(t))$ can be divided into two parts, $P_{cn_{i}}^{idl}(I_{i}(t))$ and $P_{cn_{i}}^{dny}(I_{i}(t))$. Therefore:

(6) $$\begin{array}{l} E_{cn_{i}}=\int_{t_{0}}^{t_{n-1}}P_{cn_{i}}(I_{i}(t))d_{t}=\int_{t_{0}}^{t_{n-1}}\left(P_{cn_{i}}^{idl}(I_{i}(t))+P_{cn_{i}}^{dny}(I_{i}(t))\right)d_{t}\\ \;=\int_{t_{0}}^{t_{n-1}}P_{cn_{i}}^{idl}(I_{i}(t))d_{t}+\int_{t_{0}}^{t_{n-1}}P_{cn_{i}}^{dny}(I_{i}(t))d_{t}\\ \;=\int_{t_{0}}^{t_{n-1}}U_{cn_{i}}\cdot I_{idl}d_{t}+\int_{t_{0}}^{t_{n-1}}U_{cn_{i}}\cdot i_{cn_{i}}^{dny}(t)d_{t}\\ \;=U_{cn_{i}}\cdot I_{idl}\cdot (t_{n-1}-t_{0})+\int_{t_{0}}^{t_{n-1}}U_{cn_{i}}\cdot i_{cn_{i}}^{dny}(t)d_{t} \end{array}$$

When the time interval $\left[t_{0},t_{n-1}\right]$ is divided into *n*−1 constant time interval $\left[t_{0},\;t_{1},\;\ldots ,\;t_{n-1}\right],\;\forall \left[t_{k-1},t_{k}\right],\;k\;\in \left[1,\;2,\;\ldots ,\;n\right]$, $i_{cn_{i}}^{dny}(t\in \left[t_{k-1},\;t_{k}\right])\equiv i_{ik}$, and $i_{ik}$ is a constant, then we obtain:

(7) $$\begin{array}{l} E_{cn_{i}}=U_{cn_{i}}\cdot I_{idl}\cdot (t_{n-1}-t_{0})+\int_{t_{0}}^{t_{n-1}}U_{cn_{i}}\cdot i_{cn_{i}}^{dny}(t)d_{t}\\ \;=U_{cn_{i}}\cdot I_{idl}\cdot (t_{n-1}-t_{0})+\int_{t_{0}}^{t_{1}}\left(U_{cn_{i}}\cdot i_{i_{0}}\right)d_{t}+\int_{t_{1}}^{t_{2}}\left(U_{cn_{i}}\cdot i_{i_{1}}\right)d_{t}+\cdots +\int_{t_{n-2}}^{t_{n-1}}\left(U_{cn_{i}}\cdot i_{i_{n-2}}\right)d_{t}\\ \;=U_{cn_{i}}\cdot I_{idl}\cdot (t_{n-1}-t_{0})+U_{cn_{i}}\cdot i_{i_{0}}\cdot (t_{1}-t_{0})+U_{cn_{i}}\cdot i_{i_{0}}\cdot (t_{2}-t_{1})+\cdots +U_{cn_{i}}\cdot i_{i_{0}}\cdot (t_{n-1}-t_{n-2})\\ \;=U_{cn_{i}}\cdot I_{idl}\cdot (t_{n-1}-t_{0})+\sum_{k=1}^{n-1}U_{cn_{i}}\cdot i_{i_{k-1}}\cdot (t_{k}-t_{k-1})\\ \;=U_{cn_{i}}\cdot I_{idl}\cdot (t_{n-1}-t_{0})+U_{cn_{i}}\sum_{k=1}^{n-1}i_{i_{k-1}}\cdot (t_{k}-t_{k-1}) \end{array}$$

The proof ends. ☐

**Lemma** **1.** *(Cluster energy consumption). Cluster energy consumption* $E_{clu}$ *is the quantitative denotation of the energy consumption of the GPU cluster computing environment. If the GPU cluster has n computer nodes, the cluster energy consumption* $E_{clu}$ *during time interval* $\left[t_{0},t_{n-1}\right]$ *can be quantified by Equation (8):*

(8) $$E_{clu}=\sum_{i=0}^{n-1}E_{cn_{i}}$$

Hence, we have:

(9) $$E_{clu}=\sum_{i=0}^{n-1}\left(U_{cn_{i}}\cdot I_{idl}\cdot (t_{n-1}-t_{0})+U_{cn_{i}}\sum_{k=0}^{n-1}i_{i_{k-1}}\cdot (t_{k}-t_{k-1})\right)$$

In our computing system, the voltage is a constant 12 V, and the current is measured by the homemade power consumption monitoring system of the GPU cluster.

### 3.3. Power Consumption Monitoring System Based on WSN

**Figure 5:**
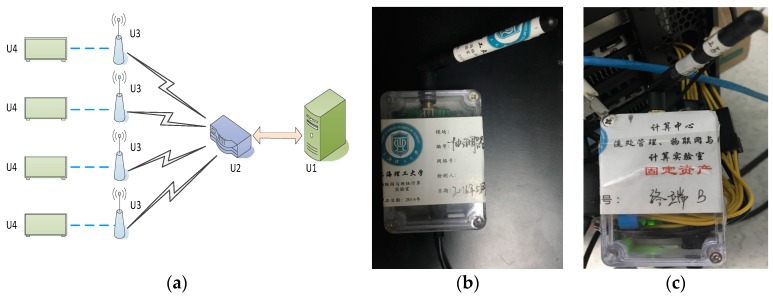
GPU energy consumption monitoring system: (**a**) The schematic diagram of the system; (**b**) The Zigbee coordinator; (**c**) A sensor node. (Note: The Chinese in caption indicates the affiliation and its identifier of this equipment.)

In Section 3.2, we discussed that the current is obtained from a homemade wireless sensor network, the power consumption monitoring system of the GPU cluster [41]. As seen from Figure 5, it is composed of multiple components: a master monitor terminal U1, a Zigbee coordinator U2, multiple sensor nodes U3 and a U4b GPU cluster (including multiple GPU computing nodes). The system also contains multiple slave monitor terminals, but these are not shown in Figure 4. U3 contains a node controller and a Hall current sensor connected to the node controller and a Zigbee communication module. For each sensor node, the Zigbee coordinator U2 is inter-connected with the master monitor terminal through the communication line, and the Zigbee communication module is inter-connected with the Zigbee coordinator using the Zigbee communication mode. The node controller is inter-connected with the slave monitor terminal through the communication line, and the Hall current sensor is set on the power supply line of each GPU, respectively.

In our sensor network, the Zigbee coordinators use the CC2530 chip to form the network and transfer packets and instructions. The sensor node is responsible for collecting the current data of the computing node in a cluster, and transfers the collected data through Zigbee to the main monitoring node, and the main monitoring node stores and displays the data in real time. The sensor node can also transfer the collected data to the sub-monitoring node, and the sub-monitoring node stores and displays the data in real time. The CC2530 chip has different operating modes, which is suitable for a system that requires low power consumption, and the time of switching between different modes is short, which further ensures low energy consumption. The model of the Hall current sensor in the sensor node is the WHB-LSP5S2H. The Hall current sensor mainly measures the current of the GPU power supply line, and transmits the measured current value to the node controller. Then, the node controller performs the analog-to-digital conversion.

Our sensor nodes have two kinds of working modes: wireless and wired. In the wireless mode, its maximum sampling frequency is 50 Hz, while in the wired mode, its maximum sampling frequency is 100 Hz. Having a sufficient number of sampling points in these two modes ensures the accuracy of the calculation result and provides an accurate basis for the evaluation of the experimental results.

## 4. Two-Level Parallelism Optimization Model

Generally, an algorithm’s performance is limited by multiple factors, such as memory/ instruction throughput, latency and so on. In previous studies, many researchers have proposed various ways to enhance the performance of the parallel algorithm. The work in [42] mainly studies the effect of warp sizing and scheduling on performance, and the work in [43] also analyzes the impact of warp-level sizing and thread block-level resource management. Both these studies adjust the number of active warps to improve performance. The work in [44] analyzes the usage of computing resources and memory resources for different applications, and it simulates the maximum TLP and exploits the underutilized resources as much as possible. ILP, which proposes to build SCs for GPU-like many-core processors to obtain both high performance and high energy efficiency is presented in [45]. The latter two methods are verified in a simulation environment. Here we combine TLP and ILP to address the performance and energy consumption problem, and we demonstrate our method in a real environment.

### 4.1. Distinguish Kernel Type

Though the NVIDIA Visual Profiler is able to analyze the limiters of the performance of parallel programs, it does not state the specify method by which to distinguish the type of kernel. Therefore, we first should determine the type of kernel according to the use of different resources by the program. We analyze the type of kernels based on two metrics: the ratio of compute utilization to hardware (HW) peak and the ratio of device memory utilization relative to HW peak. If one of the metrics is close (“close” is approximate, that is to say, 65% of theory or better) to the HW peak, the performance is likely limited by it [46]. If neither metric is close to the peak, then unhidden latency is likely an issue. We present a simple method by which to judge the type of kernel. Firstly, it is important to be aware of the related information about computing nodes at the GPU cluster, as shown in Table 2.

We can obtain the peak of memory bandwidth and maximum compute resource of different GPUs from Table 2. Then, we define $ratio_{mem}$, $ratio_{com}$, and they represent memory utilization and compute resource utilization of the GPU, respectively:

(10) $$ratio_{mem}=\;\frac{global\;memory\;utilization}{memory\;throughout\;}$$

(11) $$ratio_{com}=\;\frac{global\;compute\;utilization}{compute\;throughout\;}$$

(12) $$R=\frac{ratio_{com}}{ratio_{mem}}$$

**Figure 6:**
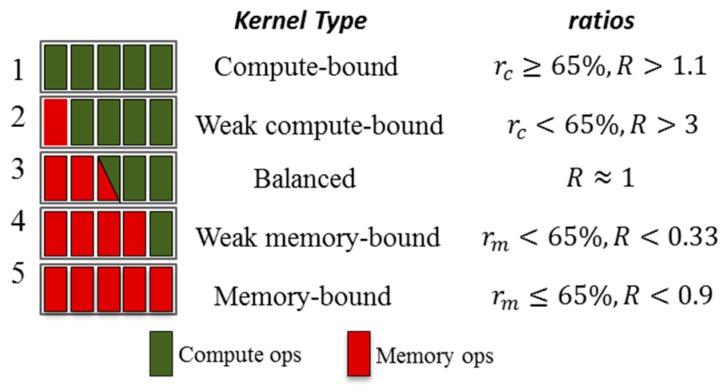
Kernel type categorization based on the resource utilization ratios. The critical values are empirically chosen.

In terms of the above rules, if either of the ratios ($ratio_{mem}$ and $ratio_{com}$) is more than 65% firstly, the kernel is likely limited by the metric. To better characterize kernel behavior, we break the kernels down into the five types, as shown in Figure 6, based on their resource utilization ratios: compute-bound, weak compute-bound, balanced, weak memory-bound and memory-bound.

The classification results not only help improve performance (as discussed in Section 4), but also provide guidance to assist system reliability (in Section 5.2). The classification results are presented in Table 1. In the next sections, for convenience, we classify weak compute-bound type and compute-bound type as compute-bound (CB) type, weak memory-bound type and memory-type as memory-bound (MB) type.

### 4.2. Coarse-Grained Thread-Level Parallelism

#### 4.2.1. The Impact of Higher TLP

GPUs exploit tens of thousands of concurrent threads to cover processing latency for arithmetic calculation and memory access. If a warp is paused by a data dependency or long latency memory access, then warp schedulers issue another ready warp from the warp pool so that the execution of warps is interleaved [44]. The availability of pause covering relies on the amount of eligible warps in the SM, which is the primary factor why GPUs require a large amount of synchronized threads [47]. Here, we use TLP to quantify the proportion of active warps in a SM. Higher TLP does not always means higher performance, however, low TLP always lacks the ability to cover memory latency, leading to performance degradation. In order to allocate the most appropriate TLP, we should adhere to the following rules.

#### 4.2.2. Appropriate Block Configuration

For the Kepler GTX 680, similar to most other GPUs, most of the key hardware units for graphics processing reside in the SM. It has 8 SMs and 192 CUDA cores per SM, the specific parameters of which are shown in Table 3.

The number of threads per block should be a multiple of 32, the size of the warp, because it makes the best computing efficiency. The dimension of blocks per grid and the dimension of threads per block have no effect on performance [33]. Hence, this section only pay attention to size instead of dimension. When considering the first configuration parameter, the number of blocks per grid, our goal is to ensure all the CUDA SMs are busy. So, the number of blocks in the grid should be larger than the number of SMs. And, there should be enough active blocks in each SM so that blocks do not have to wait for a __syncthreads() can keep the hardware busy. Another important parameter also needs to be considered, this being the number of threads per block. For TLP, we have:

(13) $$N_{warp\_block}=\frac{N_{thread\_block}}{sizeof(warp)}$$

(14) $$N_{warp\_SM}=N_{warp\_block}\times N_{block\_SM}$$

(15) $$P_{degree}=\sum_{i=1}^{n}N_{block\_SM}\times N_{thread\_block}$$

(16) $$N_{block\_SM}\leq \left\lceil \frac{MaxThreadInSM}{N_{thread\_block}}\right\rceil$$

(17) $$MinThreadInSM\leq N_{thread\_block}\leq MaxThreadInSM$$

where $N_{warp\_block}$ represents the number of warps in a thread block. $N_{block\_SM}$ represents the number of blocks in each SM, which may be not a constant for different SMs because blocks are assigned to SMs in a round-robin manner. However, no matter how the number of blocks and threads are changed, we must ensure that the total parallelism of the current program remains unchanged. As presented in Equation (15), *n* represents the number of SMs in the current GPU.

#### 4.2.3. Register Usage

We tested our GTX 680 according to the work in [48]. Accessing a register consumes about 0.19 clock cycle for a floating-point instruction, but delays may appear due to register access dependencies or register memory bank conflicts. The latency of access dependencies is about 24 cycles. And if want to completely hide the latency on SPs, it might require thousands of threads for a device of GTX 680. The number of registers used by a kernel will have a strikingly effect on the number of resident warps and register storage enables threads to keep local variables nearby for low latency access. However, the amount of register files is restricted and they are allocated to the blocks all at once, so if the per thread block uses numerous registers, the number of active blocks on a SM will be reduced.

Without registers overflowing, the maximum number of register files is 64 K per SM, and the total amount is less than the device limit. Under previous conditions, decreasing appropriately private variables in each thread can reduce register usage per thread, and thus increase the TLP per block. Using register files, we have:

(18) $$N_{register\_block}=N_{register\_thread}\times N_{thread\_block}$$

(19) $$N_{block\_SM}\leq \left\lceil \frac{RegisterFileInSM}{N_{register\_block}}\right\rceil$$

(20) $$N_{register\_thread}<64$$

when the number of private variables in a thread is much more than the register files it owns, register overflow will happen and private variables will be moved to other memory.

#### 4.2.4. Shared Memory Usage

Shared memory is high-speed on-chip memory. It is effectively a user-controlled L1 cache in Fermi and Kepler. In each SM, the L1 cache and shared memory share a 64 K memory segment. Since Maxwell, its SM unit features an independent shared memory, while its L1 cache and the texture cache have the shared memory segment. Shared memory is a block of read/write memory which can be accessed by all the threads within the same block. By facilitating inter-thread communication, shared memory enables a broad range of applications to run efficiently on the GPU. Using shared memory is the best method to achieve inter-thread communication minimum latency.

Shared memory is a bank-switched architecture. For a device of compute capability 3.x, each bank has a bandwidth of 64 bits every clock cycle. If each thread in a warp accesses the same bank address, a broadcast mechanism will be triggered to all threads within the warp, which can avoid band conflict. However, if we use other patterns, we end up with bank conflicts of varying degrees [35]. Using shared memory, we have:

(21) $$N_{block\_SM}\leq \left\lceil \frac{SharedMemInSM}{N_{sharedMem\_block}}\right\rceil$$

(22) $$N_{sharedMem\_stastic}+N_{sharedMem\_dynamic}\leq SharedMemInSM$$

when the kernel meets Table 3 and Equations (13)–(22), we can obtain an appropriate TLP configuration. For general cases, the higher TLP the programmer can achieve, the faster the program will be executed. However, sometimes if the number of active warps is limited, the ability of the SM to hide latency also drops dramatically. At some point, it will damage the performance, especially when the warp is still doing global memory accesses. If the programmer wants to increase performance further, it is recommended that larger memory transactions are made or ILP is introduced, that is, use a single thread to process multiple elements of the dataset. If we use ILP, it maximally hides latency by using ILP to increase the number of operations for every active thread. Furthermore, it makes the data acquisition operation from the global memory drop significantly, reducing the refresh rate of the memory and further lowering energy consumption.

### 4.3. Fine-Grained Instruction-Level Parallelism

Using ILP, the number of parallel instructions processed by each thread increased and the number of elements processed per thread also increased, so the number of TLP per blocks required for the same number of tasks is reduced. In per SM, the amount of blocks decreases which means that the proportion of register files, shared memory and L1 Cache that can be allocated per block is improved. At sometimes, it is likely to fully hide latency with even fewer warps, especially by ILP [49]. The detailed patterns of ILP are as follows.

#### 4.3.1. Loop Unrolling

Loop unrolling is a common compiler optimization technique that ensures there are a reasonable number of data operations for the overhead of running through a loop. In terms of the ratio of memory operations to arithmetic operations (400+ cycles for memory operations, 20 cycles for arithmetic operations), the ideal ratio that is required is at least 20:1. That is, for each memory fetch the kernel makes from global memory, it does 20 or more other instructions. However, loops without unrolling always are very inefficient because they can generate branches, and resulting pipeline stall. What’s more, they often waste instructions and do not perform any useful work. The cost of loops include: loop counter increment, loop termination test and iterative branch judgment, and computing the memory address each time.

Using loop unrolling can increase ILP and decrease the total number of iterative times. Therefore, it is able to decrease useless instructions and loop overhead. The #pragma unroll directive is provided by the NVCC compiler, which instructs the compiler to automatically unroll all constant loops.

#### 4.3.2. Using Vector Type

Using a variable of vector type simply requires declaring the variable as vector_N type, for example, int2, int4, float2, float4, because CUDA supports the built-in vector type (in vector_types.h). The programmer can also create new types, such as uchar4, and define new operators. In effect, each vector_N type is a structure which contains N elements of the base type. After using the vector type, it can increase the size of a single element, so it is able to process more than one element per thread. A certain amount of ILP can also be introduced.

Vector-based operations can allocate the cost of related operations to multiple operations rather than one, and can reduce the number of loop iterations. Correspondingly, the number of memory requests to the memory system is reduced, and the scale of request becomes larger. Using vector type will enhance register files usage per thread, which in turn reduces the amount of reside blocks for each SMX. It will further improve cache utilization, thereby reducing the global memory read/write transactions, and memory throughput will also increase as the total amount of memory transactions decline.

#### 4.3.3. Register Reuse

Register files are the fastest storage component on the GPU. Using them, the system can achieve its peak performance. However, their capacity is limited. As previously mentioned, it is better to divide a task with lots of operations into many independent parts. If so, we can use a set of registers to compute independent problems, and it is able to maximize register usage.

GPU uses a lazy evaluation model [35], only accessing memory for a variable when it needs the contents of the variable. By making the assignment and usage of a variable closer together, it enables the compiler to reuse registers. Therefore, at the beginning of the kernel, x, y and z might be assigned. But, actually, they are only used later, we can move the declaration and assignment of a register variable to the location where it can be used to realize register reuse, and thus reduce register usage. Then, the compiler is able to deal with these three independent variables being required in different and disjoint periods by simply using a single register.

By expanding the number of output data set elements of a single thread, it contributes to both MB type and CB type applications. This will increase register usage, but the number of threads being scheduled must be monitored to ensure that they do not suddenly drop off.

### 4.4. Execution Order Simulation

As an effective high-performance scheme, we propose the TLPOM. This model takes full advantage of the fact that on-chip memory resources are not completely used. Therefore, we can simultaneously use TLP and ILP technology to increase the number of instructions in each thread and decrease the total number of threads per block to avoid excessive resource competition.

**Figure 7:**
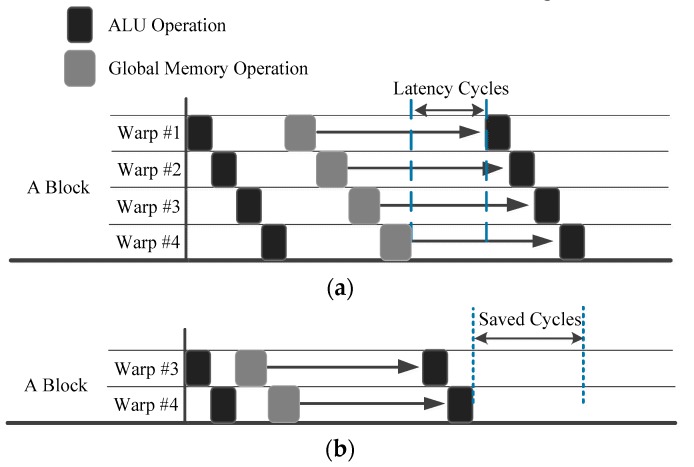
Inter-thread execution order simulation: (**a**) Execution order with ILP0; (**b**) Execution order with ${\mathrm{ILP}}_{1}=2\times {\mathrm{ILP}}_{0}$.

Figure 7 simulates the execution order of different ILPs using the TLPOM. Figure 7a presents the execution order with ILP0, and we assume for explanatory purposes that per block can issue four warps concurrently at this moment. After issuing multiple instructions from per warp in a round-robin order, each warp hits a global memory access stall [44], whereas in Figure 7b, the value of ILP1 is twice as large as ILP0, so the number of instructions of each thread per warp/block is doubled. In view of the fact that the total number of tasks has not changed, the total number of threads per block are halved.

**Figure 8:**
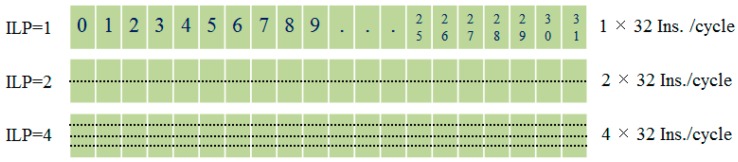
Intra-thread execution order simulation.

With our TLPOM, we reduce long latency using much less warps per block, as shown in Figure 7b. It also follows a round-robin order, but it has more memory throughput than ILP0. When executing the same number of tasks, it is able to exploit less warps (threads) which own more instructions concurrently per thread. Hence, the long latency operations can be more effectively hidden with our model. The intra-thread execution behavior is simulated as shown in Figure 8, showing the different instructions which are issued when ILP is changed for each warp per clock.

When ILP = 1, this means that every thread can only issue one instruction per cycle, and there are a total of 32 instructions in a warp; ILP = 2 means each thread can simultaneously issue two instructions per cycle, and there are a total of 64 instructions in a warp. When ILP = 4, it means a warp can issue 128 instructions per cycle. If there are sufficient ALU, Load/Store unit, SP and other resources, the more issued instructions per clock, the less time it takes to execute the same tasks. Of course, if we use varying degrees of ILP, it requires the simultaneously issued instructions to be independent.

## 5. Reliable GPU Cluster Architecture

System reliability represents the ability of the node to complete a defined function within specified conditions and a specified time. If the node fails to complete the prescribed function, it is called failure. Our computing system has multiple metrics, such as useful life, mean time between failures (MTBF) and mean time to repair (MTTF). Here we use MTBF. For repairable n products, assuming that $N_{0}$ times failures happen during execution, after being repaired, and continues to be put into use, working hours were $t_{1},\;t_{2},\;\cdots ,\;t_{N_{0}}$ of each time, respectively, so the MTBF of the product is:

(23) $$MTBF=n\cdot \frac{1}{N_{0}}\cdot \sum_{i=1}^{N_{0}}t_{i}$$

The computing system we are considered is a GPU cluster, where the GPU-enabled computing nodes can communicate through any message-passing standard, particularly the message passing interface (MPI). In the previous section, we committed to improve the algorithm’s performance for a more rapid and real-time data processing environment, but such a system is prone to failure if it runs for a long time. Hence, a problem which cannot be ignored is how to ensure the system provides 24/7 uninterrupted and high-reliability services. Hence, to achieve this we develop a reliable GPU cluster architecture (RGCA) for IoT computing.

### 5.1. GPU Cluster Reliability Scheduling Under Limited Resources

To guarantee system reliability, each computing node and its components should be assigned a proper workload to avoid overusing a component with limited GPU resources. For instance, if a task needs a large amount of shared memory but it is assigned to a GPU with a small amount of shared memory, there will be a considerable amount of data which should have been stored in shared memory which has to move to global memory, which will cause the performance to drop. Taking into account the possible constraints, it is necessary to build a model which can infer the target GPU to which a task can be assigned. The main conditions are listed as follows:

1. The time that node i has executed, which is $T_{active}(i)$;
  (24) $$T_{active}(i)=time\_now(i)-time\_start(i)$$
2. The time that node i has been idle, which is $T_{idle}(i)$;
  (25) $$T_{idle}(i)=time\_now(i)-time\_end(i)$$
3. The various memory resources belonging to node i, which is ∑R_Component(i);
  (26) $$\begin{array}{r} \sum R\_Component(i)=R\_Register(i)+R\_Shared(i)+R\_Global(i)+R\_Constant(i)\\ +R\_Texture(i)+R\_Constant\_cache(i)+R\_Texture\_cache(i) \end{array}$$
4. The different memory resources that are needed by task j, which is ∑R_task(j);
  (27) $$\begin{array}{l} \sum R\_task(j)=R\_Register(j)+R\_Shared(j)+R\_Global(j)+R\_Constant(j)\\ \;+R\_Texture(j)+R\_Constant\_cache(j)+R\_Texture\_cache(j) \end{array}$$
  Not all resources will be used by the kernels, and the resources used by different tasks are different.
5. Whether the GPU is qualified for the task or not, which can be determined by our previous research. For example, although the GTX 280 supports the atomic operation, it doesn’t support the floating point atomic operation. Hence, the optimized k-means cannot be assigned to GTX280 because it has float-point atomic add operations after optimizing.
6. All of the above relationships need to be met so that:
  (28) $$\left\{ \begin{array}{l} Z=max(MTBF(i))\\ i\in N_{j}\\ \sum R\_task(j)\leq \sum R\_Component(i)\\ T\_active(i)\leq MTBF(i)\\ T\_idle(i)\geq T\_recover(i) \end{array}\right.$$

In Equation (29), symbol i means the *i*th node in a cluster in this paper, and i ∈[1, 2, …, n] in this paper. Symbol j means the *j*th task being executed in the cluster, and j∈[1, 2, …, n]. $N_{j}$ represents the node set which contains all the available nodes for task j. MTBF(i) represents the mean time between the failure of node i, and we can obtain its value via multiple experiments and Equation (24). T_recover(i) represents the recovery time of node i, and this period ensures that the temperature of the node is returned to its idle state. The objective function Z means maximizing the MTBF for each node in the cluster. If the aim is achieved, the number of failures will be reduced which further guarantees the reliability of the computing system. The temperature is tested by GPU-Z under 26 °C. The resource required by a program must be less than the corresponding resource available to the node, such as register files and shared memory.

### 5.2. Determine Target Node for High Reliability

**Figure 9:**
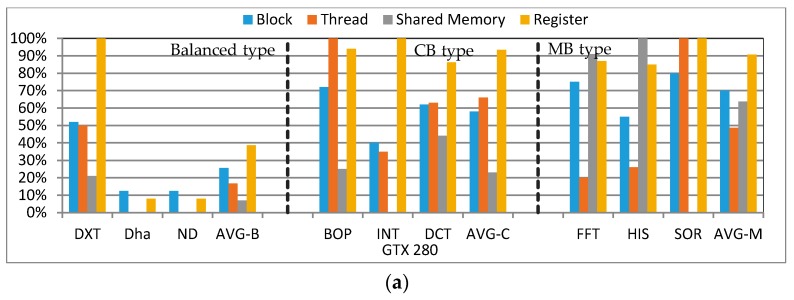

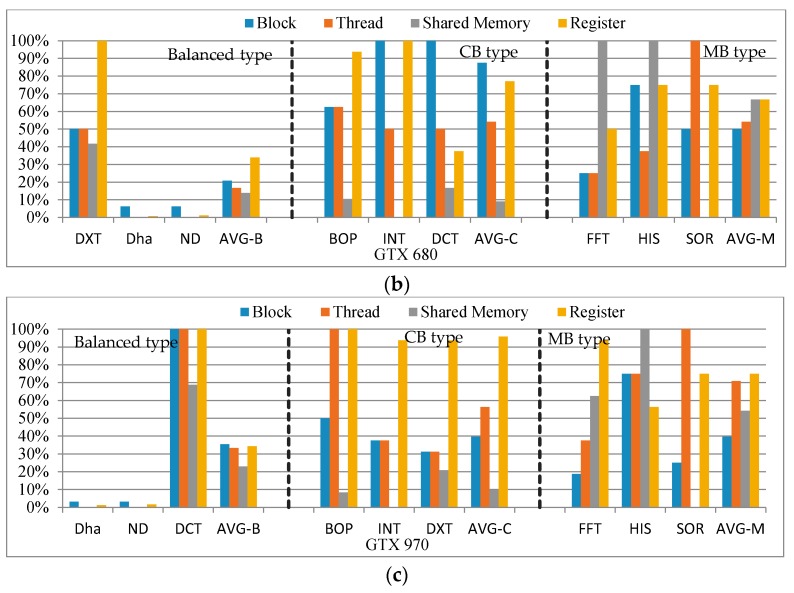
The resource utilization of different type kernels on different GPUs. (**a**) Resource utilization on GTX 280; (**b**) Resource utilization on GTX 680; (**c**) Resource utilization on GTX 970.

In addition to quantifying a variety of resources in the cluster, for different type kernels, we also need to statistically analyze the resource usage of different nodes. This analysis can assist the reliability scheduling of our cluster. We quantify four kinds of primary resources, as shown in Figure 9. If any of the resource usage reaches its limit, no more blocks can be scheduled even though all the other resources are still available. Figure 9 presents the proportion of SM resources used by primary kernel/kernels (execution duration more than 70%) of each algorithm. In Figure 9, we statistically analyze nine programs from different experiment suites, including CUDA SDK, Rodinia3.1 [50] and SHOC suite [51].

From these results, we can draw the following conclusions:

1. In terms of the balanced type kernel, for these GPUs, the utilization of four kinds of resources is relatively balanced, and the average utilization of each resource is significantly lower than the other two types of kernels. In this case, if more than one node can be assigned to the balanced type kernel according to Section 5.1, firstly we choose the lower architecture to fully exploit the resources (select 280 instead of 670/680);
2. In terms of the CB type kernel, the utilization of four kinds of resources is unbalanced. In particular, the average utilization of register files is significantly higher than other resources, and the utilization of shared memory is the lowest among them. In this case, firstly we choose the node who has the most register files available (select 680 instead of 280);
3. In terms of the MB type kernel, the utilization of four kinds of resources is also relatively balanced, but it is obviously higher than the balanced type kernel. Furthermore, both register files and shared memory have higher utilization. In this case, we select the most advanced node for the CB type kernel (select 970 instead of 680).

We also analyze our eight algorithms, and the above conclusions are also applicable to our algorithms. In order to ensure a high-performance and high-reliability computing system, we undertake a comprehensive evaluation in the following two subsections. If more than one GPU can be available for a specified task according to Section 5.1, we choose the final scheme considering the type of kernels.

## 6. Evaluation

### 6.1. Experimental Environment

The experiment platform comprises two parts: a GPU computing cluster and its WSN-based energy consumption monitoring system. The monitoring system provides a measured current to obtain the energy consumption of each node, and the primitive current value is sent to a data-log database every 10 ms (an adjustable interval). We measured the idle current and idle temperature of the GPUs, as shown in Table 4. Here, the computing cluster includes one master node and 8 computing nodes, and each is configured with a 4-core CPU, Inter® Core™ i5-3470 CPU @ 3.20 GHz, 8 G memory, 500 G hard disk, Ubuntu 14.04 OS, and the node distribution of the cluster is shown in Table 4.

**Figure 10:**
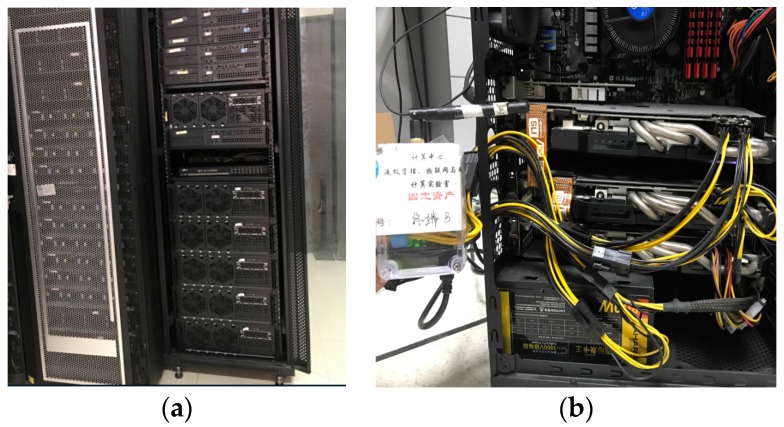
GPU cluster: (**a**) The GTX 280 and 670/680 cluster; (**b**) The GTX 970 cluster. (Note: The Chinese in caption indicates the affiliation and its identifier of this equipment.)

As shown in Table 4, the GTX 670 also belongs to Kepler. The other related information on these nodes was shown in Table 2 and Table 3. The node deployment is shown in Figure 10.

We also use the largest available input set for each application because some programs dispatch only a small amount of thread blocks with a small input set, which leads to an artificially low resource utilization.

### 6.2. Consequences

The experimental environment and related parameters set in the previous work. We first rewrite and optimize all the algorithms using our TLPOM. Without loss of generality, all algorithms are executed ten times independently to obtain the average statistical results in each experiment. The experiment is designed with considering the following aspects:

1. In a situation where the algorithms’ performance is steadily increasing, the performance of the original algorithms and our optimized algorithms after using the TLPOM are compared;
2. The data needs to be processed per second from the real-time data packets of 420,000 families, and each packet size is 125 bytes. As a result, the amount of data generated by the access system is approximately 48 M per second. In order to verify the compute capacity of our system, we increase the amount of data processed to one million packets, that is, the amount of data is 119.2 million bytes;
3. In order to guarantee the accuracy of the experiments, we set up a number of TLP and ILP. The value of TLP can be set as 32, 64, 128, 256, …, 1024 and the value of ILP can be set as 1, 2, 4, 8, …, 32. But in practice, not all algorithms can take all of these values;
4. In order to compare different performance and energy consumption behaviors of different nodes for the same application in the GPU cluster, we undertake step 1 and step 2 using three different kinds of computing nodes.

#### 6.2.1. Algorithms’ Performance Analysis on a Single Computing Node

**Figure 11:**
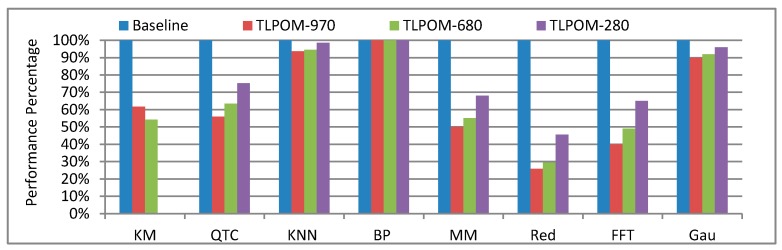
Algorithms’ performance with different GPUs.

To validate the advantages of our algorithms’ performance, we perform an exhaustive launch configuration exploration across all TLP and ILP combinations for our eight algorithms. We previously stated that the optimized k-means cannot be executed in GTX 280 because of its floating-point atomic operation. For each of these architectures, we compare the baseline performance and its optimized (TLPOM) performance. All the results are normalized relative to the baseline performance. What we need to emphasize is that the baseline performance of the same algorithm is changed along with the GPU. For statistical convenience, we only present one set of baseline performances in Figure 11, but its value is changed along with different GPUs. That is to say, the value of the optimized performance of KM in GTX 680 is not necessarily better than in GTX 970.

From the figure, it can be seen that when using TLPOM, most of the algorithms display a significant improvement in performance. In terms of GTX 970, the performance of k-means increased by 40% and Matrixmul also increased by 49.9%. For the other two GPUs, their performance also increased by different degrees. However, we observe that TLPOM is not applicable to BPNN. This is because BPNN has a lot of neurons (input layers, output layers and hidden layers) which add complexity to BPNN. Furthermore, the number of blocks and the number of threads per block are fixed, so our optimized model is not applicable to it.

**Figure 12:**
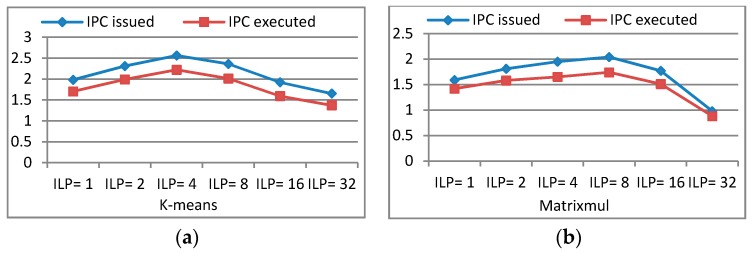
IPC issued and executed behaviors: (**a**) IPC of k-means; (**b**) IPC of Matrixmul.

In order to explore the reasons why TLPOM can deliver performance improvement, we take k-means and Matrixmul as examples for an in-depth analysis with GTX 680. Figure 12 presents the related instructions per clock (IPC) issued and the executed behaviors with different TLP and ILP. IPC means the achieved instructions throughputs per SM, and the theoretical maximum peak IPC is defined by the compute capabilities of the target device.

**Figure 13:**
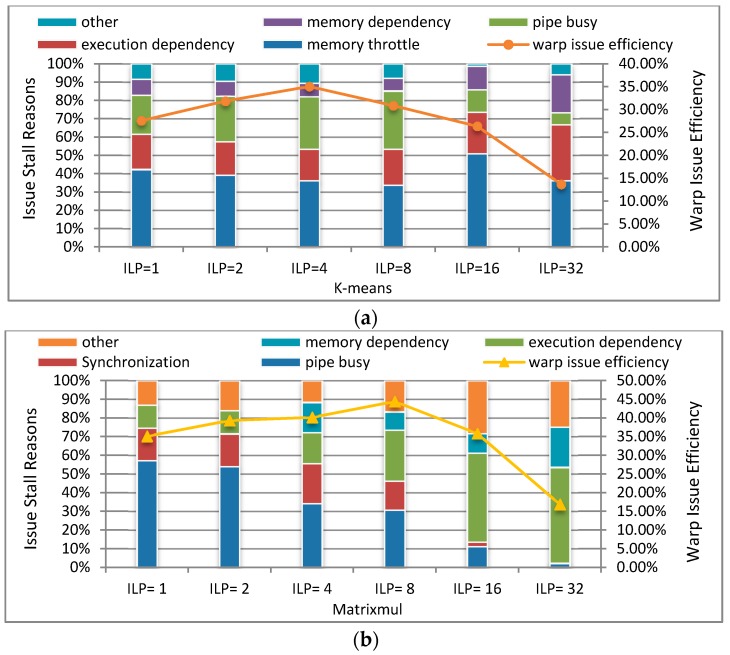
Warp issue efficiency and warp issue stall reasons: (**a**) KM warp stall reasons; (**b**) MM warp stall reasons.

With the changing of ILP (TLP is also changed), the issued IPC (the average number of issued instructions per cycle) and executed IPC (the average number of executed instructions per cycle) of the programs have corresponding changes. When the program has a higher issued IPC, more instructions can be issued per clock, so latency becomes short. A higher executed IPC indicates the more efficient usage of the available resources, and the programs have better performance. The maximum achievable target IPC for a kernel is dependent on the mixture of the instructions executed. From Figure 12, we know that k-means and Matrixmul can issue maximum instructions per clock when they have four and eight independent instructions in a single thread, respectively. At their respective highest executed IPC points, they can achieve the best performance. Another important factor in performance improvement is the change of warp issue efficiency. This represents the percentage of cycles which have eligible warps across all cycles over the duration of kernel execution. When we use the appropriate TLP and/or ILP, both the warp issue efficiency and eligible warps per clock are increased, as shown in Figure 13.

Figure 13 shows the maximum values of warp issue efficiency at the best performance point. We can see the proportion of warp issue efficiency, and there are multiple reasons resulting in a warp scheduler having no eligible warps to select from so therefore it does not issue an instruction. Figure 13a gives the warp issue stall reasons of k-means, that is, the reasons that capture why an active warp is not eligible. It is clear that the warp stall reasons change with the changeability of ILP (TLP can also be changed). Firstly, it has the highest warp issue efficiency (35.03%) when ILP = 4, as can be seen from the deputy coordinates. Secondly, the percentage of execution dependency and memory dependency are lowest at ILP = 4, and at this point, they have minimal impact on performance. Because the right combination of TLP and ILP can reduce issue stall. The data in Figure 13b further verify our views that the maximum warp issue efficiency is when ILP = 8. At this moment, the percentage of execution dependency and memory dependency are both lower than the others, so execution dependency stalls can potentially be reduced if it has proper ILP. Using loop unrolling, loading vector types are also a way to increase the scale of memory transactions. Therefore, the warp issue efficiency can be increased.

**Figure 14:**
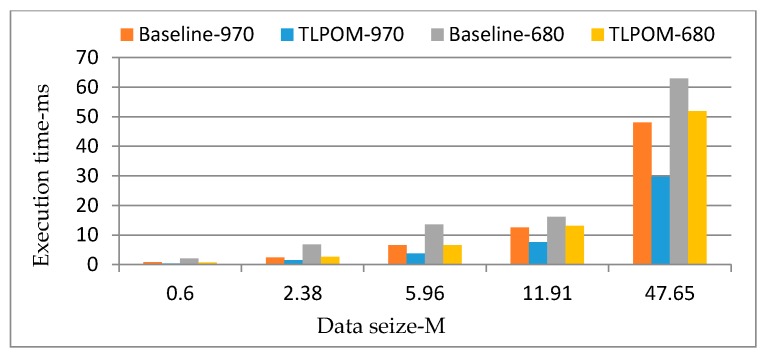
The performance of KM with different data size.

We also analyzed the performance of different amounts of data. We take k-means as the example to explain the results. As shown in Figure 14, our TLPOM works for different amounts of data for k-means, and the size of the data that needs to be processed does not affect the effect of our TLPOM. This conclusion also applies to the other seven optimized algorithms. Though the results of Figure 12 to Figure 13 are obtained in GTX 680, these conclusions are also applicable to other GPUs in our computing cluster.

#### 6.2.2. Algorithms’ Energy Consumption Analysis on a Single Computing Node

**Figure 15:**
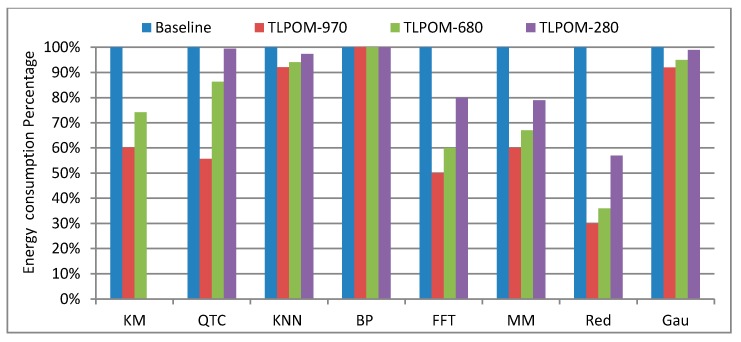
Energy consumption of data mining algorithms.

We presented the related performance results in the previous section. Here, we present the energy consumption behavior of the optimized algorithms and the results are shown in Figure 15. The results are also normalized as in Figure 11, and the values of the baseline energy consumption of the three GPUs are different. The figure presents that the energy consumption of these algorithms is almost inversely proportional to their performance. Except for BPNN, all the others have different degrees of energy consumption decline, especially Matrixmul and Reduction. Why does the energy consumption of these algorithms reduce after adjusting the TLP and ILP? Because the TLPOM increases the operations/elements of a single thread to process and also increases the register files and the usage of other on-chip memory structures. Furthermore, it makes the data acquisition operation from the global memory drop significantly, reducing the refresh rate of the device memory and further lowering energy consumption.

#### 6.2.3. Reliability Scheme of the System

In Section 5, we analyzed in detail the RGCA with *n* (*n* is a finite positive integer) computing nodes, and here we detail and verify our reliability scheme. Firstly, in order to obtain the MTBF of the cluster and consider the feasibility of the experiments, we execute three different types of kernels for 720 h (the execution time is adapted to all reliability experiments) using all the nodes in the cluster, respectively. If an application fails on a node, the node will stop to rest until it is restored to the original state. We choose three of the algorithms as representatives, the MB type program—KM (in which the KM on GTX 280 is replaced by the same type—MM), the CB type program—BPNN and the balanced type program—KNN. Table 5 and Table 6 detail the fault node information of the original algorithms and the optimized algorithms in the cluster.

The two tables show the fault information in the cluster. For example, in terms of the original k-means, the GTX 280 cumulatively fails four times during the 720-h period in the cluster. But after being optimized, the number of failures in the cluster is significantly dropped. For the optimized KM, the GTX 280 cumulatively fails three times during the experiment. Fortunately, for all of the experiments, the GTX 970 is able to operate reliably without any failures. This is because the GTX 970 was designed to offer a striking leap in energy efficiency and deliver outstanding performance while simultaneously reducing energy consumption from the previous generation.

Combining Table 5 and Table 6 and Equation (24), we can get the MTBF of the cluster, as shown in Table 7. From this, we know that the MTBF will be changed after optimizing the algorithms. For the MB type program, KM has the lowest MTBF if it is assigned to GTX 280, regardless of whether it has been optimized or not. The balanced application, KNN has the highest MTBF regardless as to what kind of GPU it is assigned. Therefore, to obtain high reliability, we reallocate these algorithms. Firstly, we assign the MB type algorithms to 970 to avoid the lower MTBF at the cluster, and then assign the balanced type algorithms to the nodes with lower compute capacity.

In Table 8, we compare the reliability of the original model with our RGCA. For Scheme 1, we use the original algorithms without any performance optimization, and these algorithms are randomly assigned to different nodes in our cluster (we allocate them in the order of the first letter of the algorithms). In this case, the cluster cumulatively fails four times during the experiment. But for the RGCA system, we have strict distribution standards according to Section 5. Here we only list two of them. In this case, Schemes 2 and 3 each fail twice. Compared to the original Scheme 1, the number of failures dropped by 50% in our two reliability schemes. This is a significant reliability improvement.

However, a non-ignorable problem is that the GTX 280 causes the node to fail in Schemes 2 and 3. This is because the compute capacity of the GTX 280 is only 1.3, and its compute resources and memory resources are lower than the other GPUs in the cluster. Fortunately, through our RGCA, we can reduce the node failure rate. So we can use the lower GPU to reduce costs and save computing power in our cluster. Finally, for different types of data mining algorithms of IoT computing, we can obtain a low-cost, high-performance and high-reliability computing system by using the heterogeneous cluster.

## 7. Conclusions

The Data Access System for the Internet of Things (DASIoT), as a crucial part of the WSN, plays a decisive role in large-scale sensor data access and processing. To cope with the massive original access data in the IoT, one of the most important technologies is data mining, because it can convert the data collected by DASIoT into useful information and further provide a better service for the user.

In this paper, our goal is to provide a low-cost, high-performance and high-reliability computing system for common data mining algorithms in IoT computing. Our optimization scheme is divided into three parts. Firstly, we present an energy consumption calculation method (ECCM). Then, we provide a two-level parallel optimization model (TLPOM)-based CUDA programming and to improve the performance of these algorithms. Finally, in order to obtain a long-term, error-free runtime environment, we put forward a reliable GPU cluster architecture (RGCA) under limited resources. In the process of optimizing performance, we assign a different number of blocks/threads per grid/block to acquire the best launch configuration, and we also use common compiler optimization techniques, for example loop unrolling. On the basis of the performance improvements, we define the GPU cluster energy consumption calculation model by capturing the real-time current to calculate the energy consumption. Then, with full consideration of the computational capacity, GPU resources and the characteristics of algorithms, we obtain a high-reliability computing system. Our experiments demonstrate the effectiveness of our optimization scheme.

Further work will cover an extension of the proposed high-reliability computing system, and on this basis, we will study in-depth the reliability scheduling and fault tolerance scheme for IoT computing. During implementation, the fault detection and recovery mechanism will be added to the task execution. The fault nodes should be migrated and revisited to ensure the reliability of the system. Further work will also take into consideration the GPU computing cluster to undertake large-scale real-time data processing.

## Figures and Tables

**Table 1:** The benchmarks.

Algorithm	Application	Type	Benchmark
k-means	Clustering	M	Rodinia
QT (Quality Threshold) Clustering	Clustering	B	SHOC
KNN	Classification	B	Rodinia
BPNN	Classification	C	Rodinia
FFT	Basic Function	C	SHOC
Matrixmul	Basic Function	M	CUDA SDK
Reduction	Basic Function	M	CUDA SDK
Gaussian	Basic Function	B	SHOC

**Table 2:** Resource distribution of different GPUs.

GPU	Architecture	Register Files	Shared Memoey	Memory BW	Instrustions/Clock	CUDA Cores (Stream Processors)
GTX 280	Fermi	16 K * 32-bit	16 K	142 GB/s	1024	240
GTX 680	Kepler	64 K * 32-bit	48 K	192 GB/s	2048	1536
GTX 970	Maxwell	64 K * 32-bit	48 K	224 GB/s	2048	1664

**Table 3:** Basic parameters according to NVIDIA.

Variable	Fermi-280	Kepler-680	Maxwell-970
SM	15	8	13
CUDACoreInSM	32	192	128
MaxBlockInSM	8	16	32
MaxwarpInSM	48	64	64
MaxthreadInBlock	1024	1024	1024
MaxThreadInSM	1536	2048	2048
MaxRegisterInThread	32	64	64

**Table 4:** GPU Cluster node configuration information.

Node	Number	Idle Current (A)	Idle Temperature (°C)
GTX 280	2	2.651	56
GTX 670	1	1.949	41
GTX 680	3	1.934	40
GTX 970	2	1.065	36

**Table 5:** Fault node information of original algorithms.

GPU/Algorithms	k-means	BPNN	KNN
GTX 280	4	3	3
GTX 670	1	1	0
GTX 680	1	0	0
Sum (times)	6	4	3

**Table 6:** Fault node information of optimized algorithms.

GPU/Algorithms	k-means	BPNN	KNN
GTX 280	3	2	2
GTX 670	1	0	0
GTX 680	0	0	0
Sum (times)	4	2	2

**Table 7:** The MTBF of different types of applications in the cluster.

Algorithms (Type)	k-means (Mem-Bound)	BPNN (Com-Bound)	KNN (Balanced)
Original MTBF (h)	960	1440	1920
TLPOM MTBF (h)	1440	2880	2880

**Table 8:** Reliability contrasted against the baseline model.

Scheme/GPU		280	280	670	680	680	680	970	970
Original	Scheme 1	BPNN	FFT	Gau	KM	KNN	MM	QTC	Red
	Fault node (times)	2	2	0	0	0	0	0	0
RGCA	Scheme 2	QTC	Gau	KNN	FFT	Red	BPNN	MM	KM
	Fault node (times)	1	1	0	0	0	0	0	0
	Scheme 3	KNN	QTC	Gau	FFT	Red	MM	BPNN	KM
	Fault node (times)	1	1	0	0	0	0	0	0
